# Quantitative perfusion imaging from non-contrast micro-ct for pulmonary embolism evaluation in preclinical models

**DOI:** 10.1088/1361-6560/ae8126

**Published:** 2026-07-03

**Authors:** Blake Evans, Hsu-Ting Kuo, Joshua Joseph, Allan John R Barcena, Marvin R Bernardino, Dominic Karl M Bolinas, Steven Y Huang, Lili Zhao, Girish Nair, Marites P Melancon, Edward Castillo

**Affiliations:** 1 Department of Biomedical Engineering, The University of Texas at Austin, Austin, TX, United States of America; 2 Department of Interventional Radiology, The University of Texas MD Anderson Cancer Center, Houston, TX, United States of America; 3 Division of Biostatistics, Department of Preventive Medicine, Northwestern University, Chicago, IL, United States of America; 4 University of Kentucky College of Medicine, Lexington, KY, United States of America

**Keywords:** pulmonary embolism, computed tomography, CT-perfusion, pulmonary perfusion

## Abstract

*Objective.* Pulmonary embolism (PE) has a high mortality rate, making early diagnosis critical for patient survival. While computed tomography (CT) pulmonary angiography effectively aids diagnosis and embolus localization, patients with iodine allergies and kidney disease experience delayed diagnosis and/or adverse effects. This exploratory preclinical study qualitatively describes PE-induced hemodynamic changes observable in CT perfusion imaging (CT-P), a computationally derived surrogate for perfusion imaging derived from non-contrast four-dimensional CT (4DCT), in a small pilot cohort. *Approach*. Two laboratory mice were imaged with a micro-4DCT scanner before and after the introduction of a synthetic PE, created using radiopaque alginate beads to enable embolus localization in 4DCT images. CT-P images were computed by segmenting the right and left lungs at the maximum inhale/exhale phase and quantifying the local tissue displacements using deformable image registration. Pre- and post-embolism CT-P heterogeneity was compared as a function of proximity to the embolism in the axial, coronal, and sagittal planes. CT-ventilation (CT-V) images were also computed using the Integrated Jacobian method to investigate co-localized ventilation-perfusion changes. *Main results.* In this two-animal pilot study, pre- and post-embolism imaging enabled observations not feasible in human studies. CT-P was observed to increase locally at the embolism site but decreased distally within the same lobe. Other lobes within the affected lung showed reduced perfusion, while the contralateral lung showed a compensatory increase mirroring the embolism location. Local perfusion variance near the embolism appeared greater in the unaffected regions. A lobe-wise CT-V/CT-P mismatch of 12.79% and 13.60% was observed pre- and post-embolization. *Significance.* As a hypothesis-generating preclinical study, these results demonstrate the methodological potential of CT-P to capture embolism-associated hemodynamic changes, motivating future studies with larger sample sizes.

## 1. Introduction

Pulmonary embolism (PE) is a life-threatening condition with high mortality rates that occurs when an embolus blocks blood flow to the lungs, leading to sudden morbidity or death (Peracaula *et al* 2024). Timely diagnosis and intervention have been shown to reduce the short-term mortality rate from 33% to 8% (Bĕlohlávek *et al* 2013). However, PE is difficult to diagnose due to non-specific signs and symptoms, which often overlap with other cardiovascular diseases like pneumonia, myocardial infarction, and pleuritis (Swan *et al* 2019). Planar ventilation/perfusion (V/Q), computed tomography pulmonary angiography (CTPA), and single photon emission computed tomography (CT) V/Q scan (SPECT V/Q) are three validated non-invasive imaging modalities for PE diagnosis (Le Roux *et al* 2018, Le Pennec *et al* 2024).

CTPA is the current gold standard for PE diagnosis (Zantonelli *et al* 2022, De Jong *et al* 2024). Although CTPA is robust and readily available in most institutions, it uses intravenous iodinated contrast agents and thus exposes patients to the risks of contrast-induced nephropathy, especially patients with acute kidney failure or chronic renal insufficiency (Sedhai *et al* 2025). Additionally, patients with allergic-type reactions to iodine face major discomfort and delayed PE diagnosis. The incidence of immediate reactions to nonionic contrast media ranges from 0.01%–0.04% (severe) to 3% (mild), indicating that up to 3% of the population will have either delayed or suboptimal access to CTPA to diagnose life-threatening PE (Baerlocher *et al* 2010).

While patients who have prior allergic-like reactions can receive a CTPA following premedication, it has been reported that the current premedication pipeline in the Emergency Department (ED) significantly delays PE diagnosis (Berlyand *et al* 2022). The order to start time for allergic patients (296 min) is more than twice longer that of the non-allergic patients (118 min) (Berlyand *et al* 2022). Considering the severity of ED crowding in the United States, its known negative effects on patient care, and the importance of fast and accurate PE diagnosis, there is a significant clinical need for a non-contrast alternative PE diagnostic method. Replacing CTPA with a non-contrast imaging modality will significantly decrease the disparity in time to diagnosis for PE patients with kidney failure and contrast allergies. Alternative PE diagnostic techniques, such as SPECT V/Q, are not broadly accessible nor readily available for emergency situations.

Non-contrast CT has been explored as a more accessible alternative for PE diagnosis (Sun *et al* 2014, Elia *et al* 2021, Guo *et al* 2024). However, prior studies have reported low sensitivity and specificity when using conventional non-contrast CT for PE detection (Sun *et al* 2014). In addition, existing non-contrast CT-based approaches primarily rely on subjective visual features, such as hyperdense lumen sign, rather than quantitative measures of embolism-associated physiological changes (Tatco and Piedad 2011, Hassan *et al* 2021). Together, these limitations highlight a critical unmet need for non-contrast-based approaches capable of assessing embolism-induced hemodynamic changes.

Four-dimensional computed tomography (4DCT) is a non-contrast imaging technique that captures a series of 3D lung CT scans over the respiratory cycle (Yamamoto *et al* 2011, Willmann *et al* 2022). 4DCTs can be acquired using standard CT scanners available in EDs and have been routinely utilized in radiation oncology for planning radiotherapy treatment, showing that it is a well-established and accessible imaging modality feasible for emergency situations. In our prior work, we developed image-processing-based approaches to quantify pulmonary ventilation and perfusion from dynamic non-contrast CT scans (4DCTs) (Myziuk *et al* 2019, Castillo *et al* 2021).

CT-perfusion imaging (CT-P) quantifies subtle spatial changes in pulmonary blood mass between inhale and exhale CT scans, which serve as a surrogate for blood flow and have been shown to reflect perfusion defects associated with PE (Mirsadraee *et al* 2016, Nakamura *et al* 2024). In our previous work, we demonstrated a robust, computationally efficient CT-P algorithm that has a good correlation with SPECT-perfusion (Castillo *et al* 2021). We also demonstrated that pulmonary blood mass change is lower in PE-positive patients, suggesting the feasibility of CT-P-based diagnosis (Nair *et al* 2021). Furthermore, our team developed a deep-learning PE diagnosis model using 4DCT scans from 129 patients with suspected pulmonary emboli as a pilot study, achieving an accuracy of 0.72 (Kuo *et al* 2026). However, these models only classify patients as either PE-positive or -negative. *The purpose of this study is to identify if CT-P imaging captures changes associated with acute PE and to characterize regional perfusion heterogeneity caused by PE.* We employ micro-4DCT imaging to compute CT-P before and after synthetically induced PE within a mouse model. The synthetic PE is created using radiopaque beads, such that the location of the embolism can be visualized in a non-contrast CT scan. We demonstrate that PE-induced variations in blood mass dynamics can be captured by CT-P imaging, and we describe the implications of our novel approach to studying PE.

## 2. Methods

### 2.1. Overview

**Figure 1.**
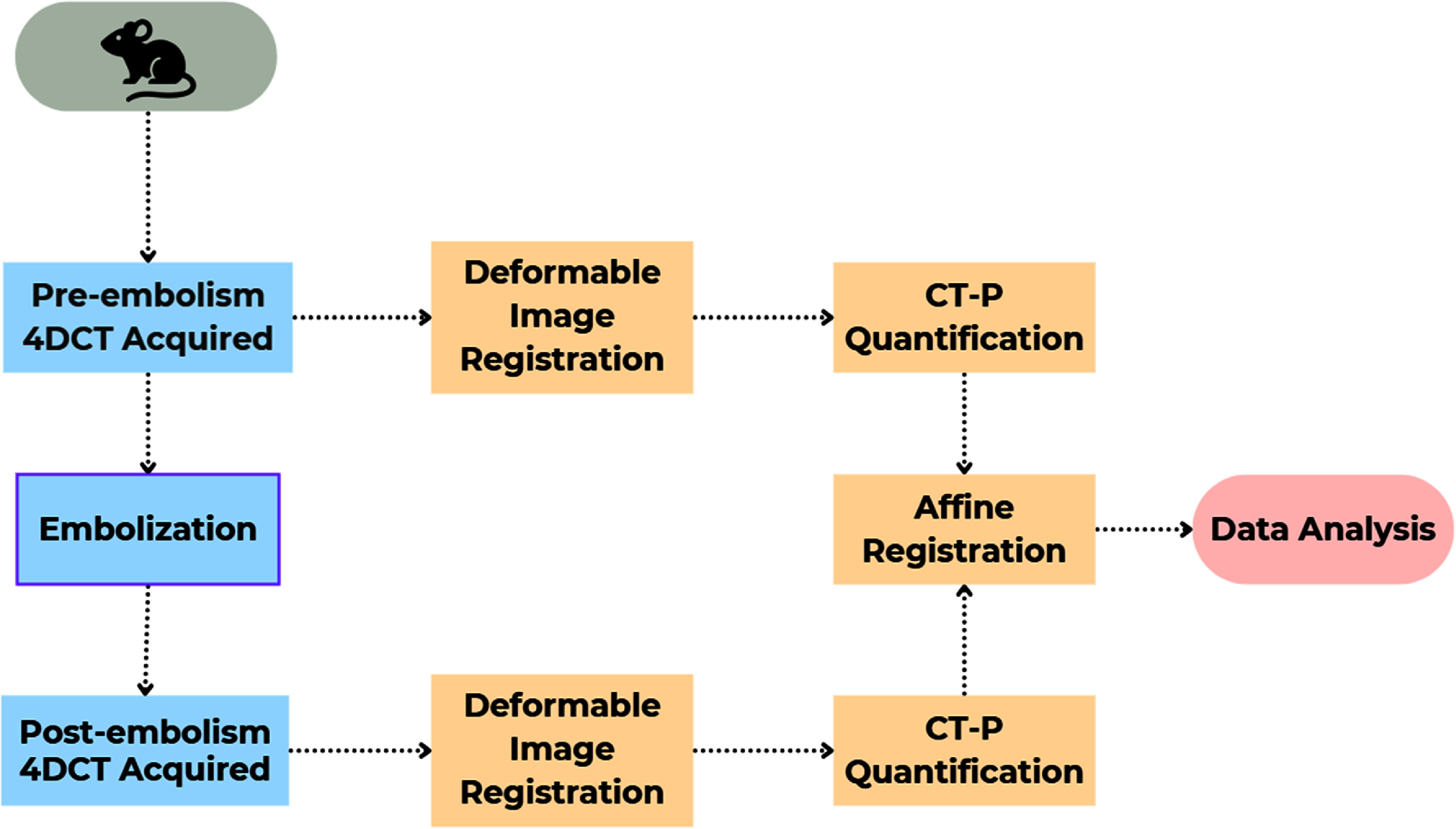
Overview of the study. The first segment (blue boxes) focuses on the animal model, including mouse embolization and 4DCT imaging. The second segment (orange boxes) focuses on image preprocessing steps, including image registration and computation of CT-P images.

Two animals were used in this pilot study. As illustrated in figure 1, the study workflow involves the animal model, micro-4DCT imaging, and CT-P computational analysis. 4DCT images were acquired before and after injecting a synthetic PE into the animal. In total, four pairs of 4DCT images were used in perfusion quantification: pre-embolization inhale/exhale pairs and post-embolization inhale/exhale pairs. The inhale and exhale images were registered using the quadratic penalty deformable image registration algorithm (Castillo 2019), and the resulting displacement vector field was used to compute CT-P images, as described in prior work (Castillo *et al* 2021). We applied affine registration to compare the differences between pre-and post-embolization CT-P, followed by further data analysis to characterize the emboli-induced perfusion changes.

### 2.2. Fabrication of radiopaque alginate beads

Sodium alginate (CAS No. 9005-38-3, Sigma-Aldrich, St. Louis, MO) was dissolved in sterile water to prepare a 2% w/v alginate aqueous solution. Then, the solution was filtered and electrosprayed at 0.1. ml min^−1^ and 6 kV using a Spraybase electrospinning system (Avectas, Maynooth, Ireland) into a 100 mM gadolinium (III) chloride (CAS No. 10138-52-0, Sigma-Aldrich) solution. This results in the cross-linking between alginate and the gadolinium ions, forming 1-mm radiopaque hydrogel beads (Melancon *et al* 2025). Gadolinium (Gd, *Z* = 64) is a high-*Z* element primarily utilized as a contrast agent in magnetic resonance imaging (MRI), but it can also be used in CT (FitzGerald *et al* 2016). Following electro-spraying, the beads were incubated for further cross-linking for 10 min, washed by filtration with 0.9% sodium chloride solution, and then air-dried for 24 h before surgical injection.

### 2.3. Induction of PE

All mouse experiments were approved by the Institutional Animal Care and Use Committee (Protocol No. 00001234). Mice were housed in a centralized animal facility in the Department of Veterinary Medicine under the jurisdiction of the Institute of Animal Care and Use Committee and in compliance with the United States Public Health Service Policy on Humane Care and Use of Laboratory Animals. This study used three 8 week-old female C57BL/6 mice (400 SAS SD, Charles River Laboratories, Wilmington, MA). The neck was dissected to expose the external jugular vein. In each mouse, one radiopaque bead was inserted into the external jugular vein and allowed to flow into the pulmonary circulation. All the animals were given appropriate analgesia and monitored for adverse reactions. Our original cohort included three female C57BL/6 mice. Pre-embolization CT imaging showed that one mouse included in the study had fluid in its right lung inferior lobe. This subject was not included in our analysis as our CT-P quantification method follows an assumption that parenchyma can be modeled as a linear combination of air and blood (Hoffman 1985, Hoffman *et al* 2003, Yin *et al* 2009).

### 2.4. Image acquisition

The Skyscan 1276 CMOS micro-CT system was used to perform non-contrast CT (Bruker, Billerica, MA). The mice were placed under general anesthesia with inhaled isoflurane (1%–2%) before imaging. CT images were obtained before and after surgical injection of radiopaque beads. Scans were acquired with a 70 kV x-ray source, 200 *µ*A current, and a 0.5-mm aluminum filter. The acquisition parameters were set to a 30 *µ*m resolution, 1008 × 672 image matrix, 148 ms exposure time, a 0.8° rotation step, and time-resolved scan with 25 Nimg.

Following listmode sorting with DataViewer (Bruker) and image reconstruction with NRecon (Bruker), CTAn image analysis software (Bruker) was used to process the images, measure the radiopacity of the grafts in Hounsfield units (HU), and visualize the grafts with grayscale palette adjustment with brightness level set at 0–100.

### 2.5. Computational pipeline

All computational methods in this study require accurate segmentation masks of the right and left lungs. Segmentation masks were manually drawn using ITK-Snap and verified by a clinician (Yushkevich *et al* 2006). We used the quadratic penalty method for deformable image registration (QPDIR) to register inhale/exhale 4DCT phases (Castillo 2019). QPDIR builds on conventional block-coordinate descent algorithms by including an equality-constrained smoothness penalty into a least-squares optimization problem. We approximated the Jacobian of the displacement field using the integrated Jacobian formulation to calculate breathing-induced volume changes. These three components (segmentation mask, displacement field, and DIR Jacobian) are used to formulate CT-P quantification as a least squares problem over computational subdomains, whose solution is the voxel-wise mass change from inhale to exhale 4DCT image phases.

We computed the CT-P images using the mass change formulation previously published (Castillo *et al* 2021), in which a parameter sweep was performed to evaluate the robustness of the approach to variations in image registration. The CT-P solution represents the change in blood mass distribution between inhale and exhale, which has been shown to correlate with pulmonary perfusion in humans. It is assumed that lung tissue can be modeled as a linear combination of air and blood (Hoffman 1985, Hoffman *et al* 2003, Yin *et al* 2009). Under this assumption, the CT-P method calculates pulmonary blood mass changes derived from inhale/exhale CT pairs as a surrogate for perfusion, using CT HUs, segmentation masks, and the displacement vector field. One mouse was excluded from the study due to a significant level of fluid observed in the pre-embolization CT image, leaving a final sample size of two. The presence of fluid in the lung violated the core assumption that parenchymal density can be approximated as a linear combination of air and blood, making CT-P computation unreliable for that subject.

While our CT-P approach is validated in human cases by comparison to SPECT perfusion imaging, the assumptions and mathematical approach hold true in animal models. Further, the micro-4DCT imaging incorporated in this study has higher spatial resolution than clinical CT imaging used to validate CT-P. CT-P images are constructed by dividing the computational domain, in this case either the right or left lung, into subdomains and solving a series of least squares problems that identify small changes in mass between spatially corresponding locations within the inhale and exhale images.

The exploratory nature of this study and the isotropic micro-4DCT voxels allowed for the computational domain to be at full resolution without any image reshaping. In our original CT-P implementation, outlier detection prevented the inclusion of vasculature and airways from the mass-change calculation, as these image regions are not representative of parenchymal perfusion. In this study, we additionally removed the embolism from the computational mask, as the relatively large HUs of the embolizing agent skewed outlier detection that removes vasculature and artifacts from the computational domain. To ensure a fair comparison, we removed an analogous region from the computational mask of the pre-embolized images as identified by affinely registering post-embolism to pre-embolism images.

We finally compared the blood mass dynamics captured by our CT-P images before and after each mouse received a surgically implanted embolus. The mice did not experience major pulmonary structural changes between each imaging study, as they occurred within a roughly 15 min window; however, variations in breathing patterns and orientation in the CT scanner resulted in misaligned CT-P images before and after surgery. The radiopaque beads used to induce the emboli produce abnormally high HU, which fall outside the range expected under the assumption that parenchymal density can be approximated as a linear combination of air and blood. Therefore, the emboli were excluded from CT-P computation, consistent with standard practice of excluding non-parenchymal structures, such as vessels and airways. To visualize the spatial difference between pre- and post-embolism CT-P images, we identified an affine transformation that mapped the pre- and post-4DCT inhale phases and applied that transformation to the pre-embolism CT-P image. This enabled a voxel-wise visualization of the changes in blood mass dynamics resulting from acute PE with the post-embolism scan as the reference geometry (see figure 3).

To consider the volumetric perfusion heterogeneity, we quantified the average perfusion radially outward from the embolism by quantifying the average perfusion in a rectangular window as a function of distance from the embolism (see figure 4). The rectangular window was defined as the smallest rectangular prism that encapsulated the embolism. The directions for computing radial changes in perfusion are Cartesian coordinates (*x, y*, and *z*), which correspond to coronal, sagittal, and axial anatomical directions, respectively. These directions were chosen for optimal interpretability and accuracy, as they follow focal planes of the high-resolution micro-4DCT imaging.

Lastly, we explored whether our CT-P method shows potential to capture lobe-level changes associated with PE. To this end, we computed CT-ventilation images (CT-V) using the integrated Jacobian method (Castillo *et al* 2019). Similar to our CT-P methodology, CT-V uses the deformation vector field from inhale- and exhale-CT scans to approximate the voxel-level ventilation throughout the lungs. Our motivation was the conventionally deployed V/Q mismatch in SPECT PE diagnosis. Lung lobes were manually segmented using ITK-Snap and verified by a clinician (Mirsadraee *et al* 2016). Lung lobe segmentations were only used for localizing global lung CT-P and CT-V to specific lobes. Mean lobe-wise CT-P and CT-V values were quantified as the average intensity per unit volume of a lobe. Lobe-wise V/Q was the ratio of the mean ventilation to perfusion, which was used to quantify the percent change in V/Q before and after induced-PE. All computations were performed using MATLAB 2025b (MathWorks, Natick, MA) on a system with Intel Xeon(R) W-2295 CPU (Intel, Santa Clara, CA).

## 3. Results

### 3.1. PE was successfully induced using radiopaque beads

**Figure 2.**
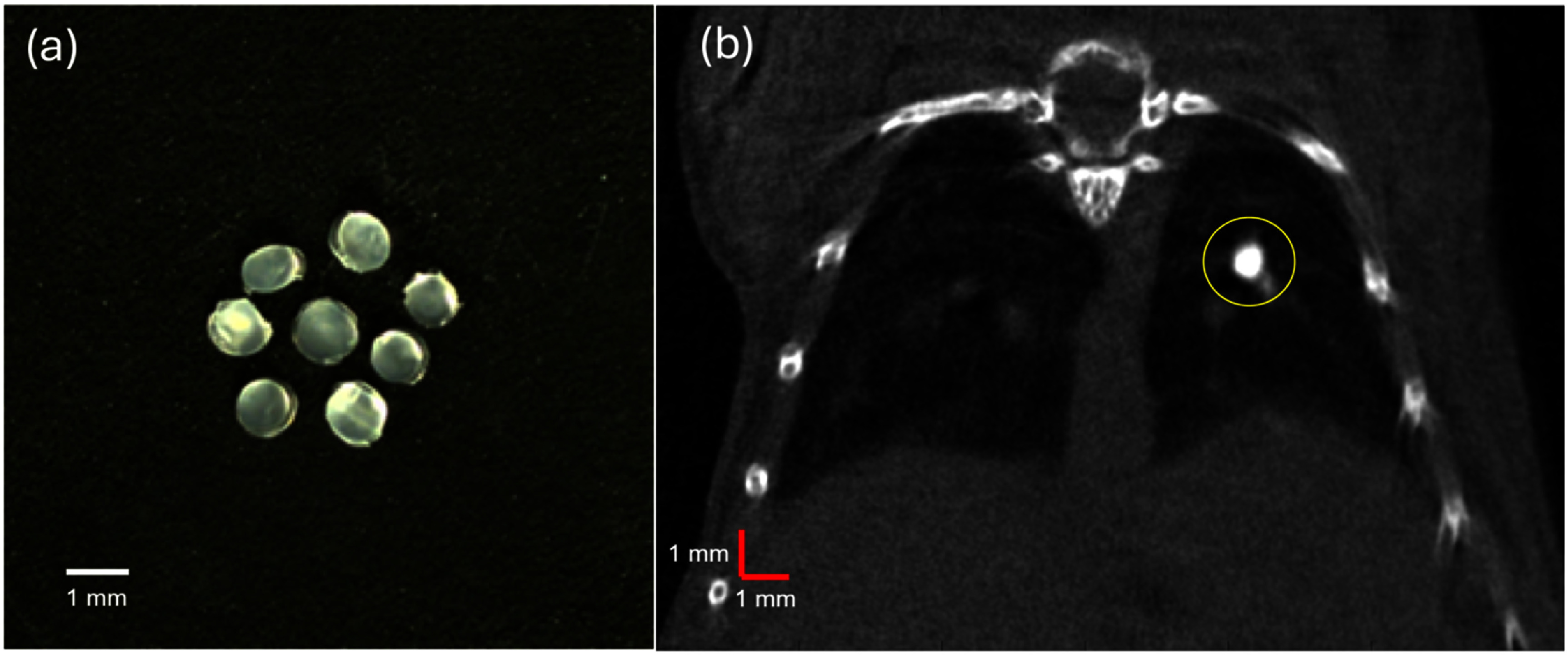
(a) Radiopaque beads and (b) 4DCT inhale image of Case 1 with a visible embolus (marked in yellow circle) in the left lung.

Electrospraying followed by a 24 h air-drying period yielded beads that were structurally stable for surgical manipulation (figure 2). Following surgical injection via the external jugular vein, the beads remained intact and successfully migrated into the vasculature to lodge within the lung, effectively simulating a PE.

Quantitative analysis of the scans validated the high radiopacity of the synthetic emboli. The beads demonstrated a mean radiodensity of 1200 HU, facilitating clear differentiation from the surrounding soft tissue, which measured approximately −320 HU, and the air within the lung parenchyma, which measured −740 HU. Notably, the beads’ radiopacity was comparable to that of the rib bone, which measured 1195 HU.

### 3.2. Quantitative perfusion imaging

**Figure 3.**
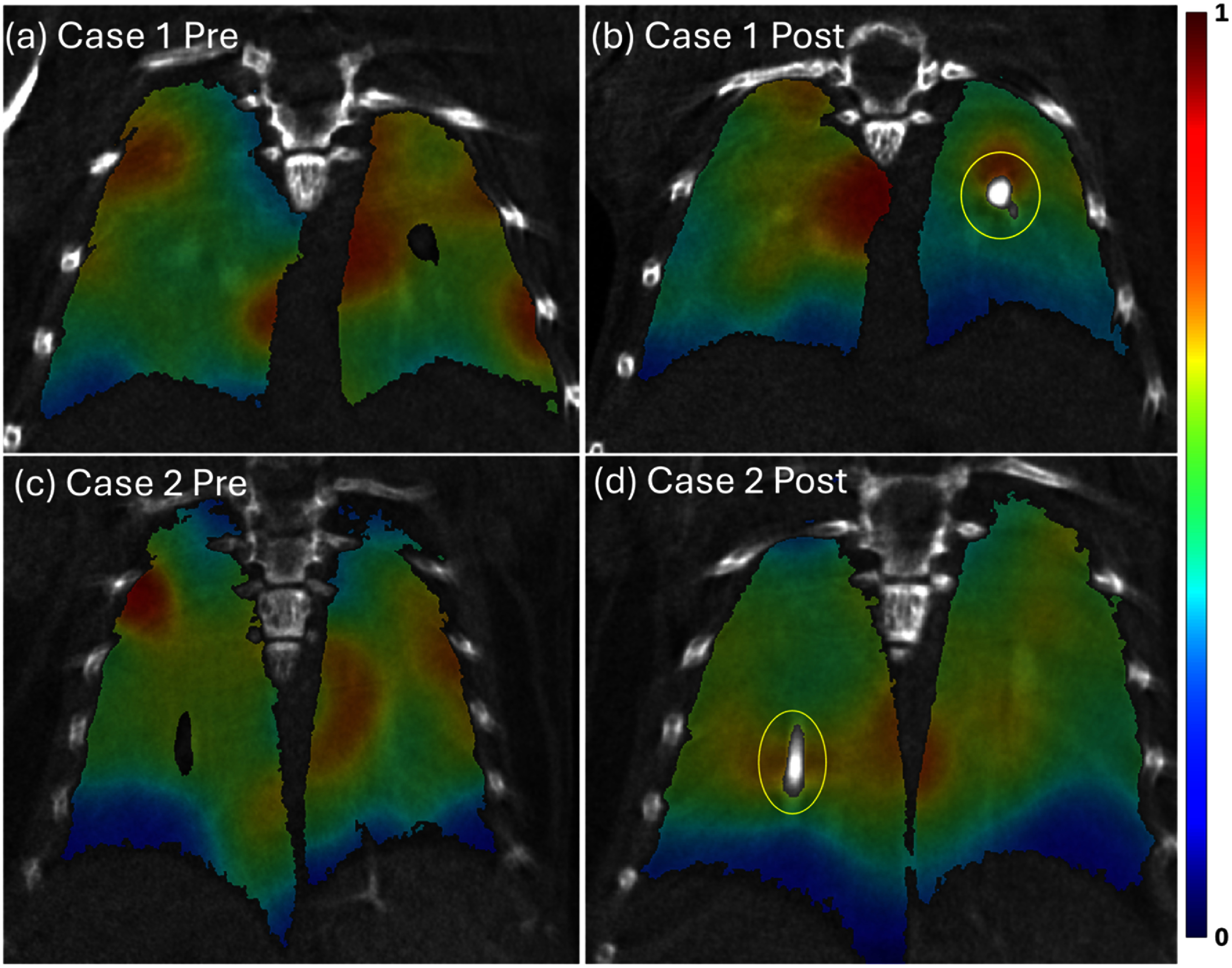
Pre- and post-embolization CT-P maps for Case 1 (a) and (b) and Case 2 (c) and (d), shown as representative coronal slices. Perfusion defects are visible in the post-embolization maps (b) and (d). Yellow circles indicate embolus locations, and the corresponding regions in the pre-embolization maps were excluded for fair comparison. The intensities were normalized within each case across the pre- and post-embolization images to enable direct visual comparison. The colorbar represents normalized CT-P values, where red indicates high perfusion and blue indicates low perfusion.

As illustrated in figure 3, the CT-P maps characterize magnitude blood mass changes occurring between the inhale/exhale scans, before and after embolization. The hot spots (red area in figure 3) indicate high estimated perfusion, and cold spots (blue area in figure 3) represent low estimated perfusion. Case 1 shows no apparent defect in its pre-embolization map, whereas Case 2 shows a pre-existing perfusion defect, which became more obvious after introducing the synthetic PE. The embolus also altered the spatial distribution of low perfusion in Case 2, with a cold spot emerging downstream of the embolus in figure 3(d).

### 3.3. Visualization and characterization of emboli-induced perfusion changes

**Figure 4.**
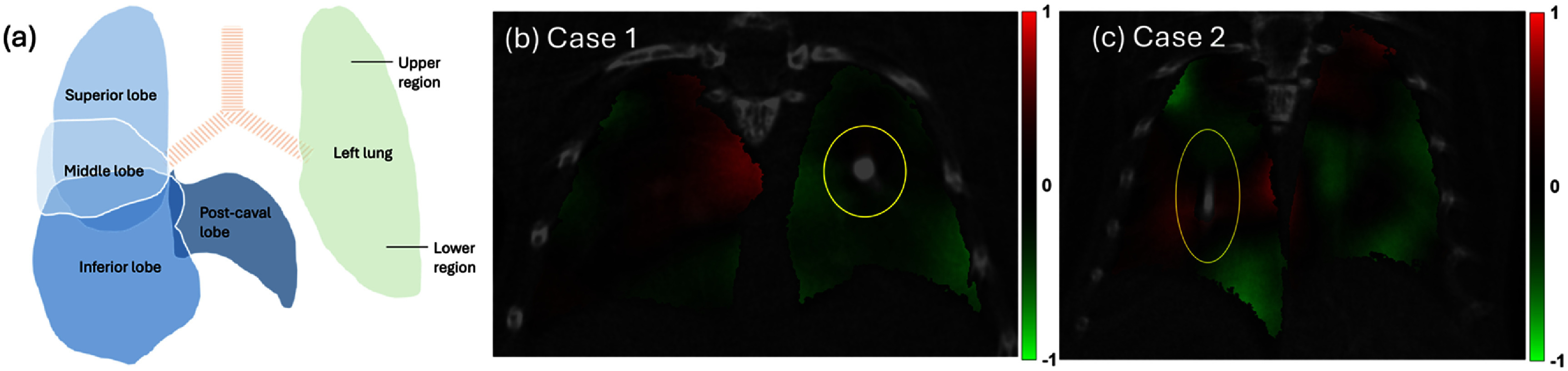
Embolization-induced changes in CT-P, shown as representative coronal slices for Case 1 (b) and Case 2 (c). (a) Schematic of the mouse lung, showing the structure of lobes for each lung. The embolus locations are indicated by yellow circles. The colorbar represents normalized differences in CT-P values between pre- and post-embolization images, where red indicates increased perfusion, green indicates decreased perfusion, and black denotes no change.

To better characterize the effects of the embolus, we used affine registration to align the pre-procedure CT-P image with the post-procedure inhale scan and visualized the differences between pre- and post-procedure CT-P (figure 4). The bright spots marked by yellow circles are emboli. For Case 1, the embolus is in the left lung, where in mice, there is only one lobe. As shown in figure 4(b), the embolus led to increased CT-P in the upper-right area and decreased CT-P in the lower-left region. This suggests blood accumulation upstream of the embolus and a reduced flow downstream, consistent with an obstruction of the vasculature supplying the inferior region of the mouse left lung, which is the more dependent segment in supine position (Phillips *et al* 2017). For Case 2, we can see the embolus in the right lung, where in mice there are four lung lobes. Figure 4(c) shows that the obstruction likely impacted the blood supply to both the post-caval and superior lobes.

**Figure 5.**
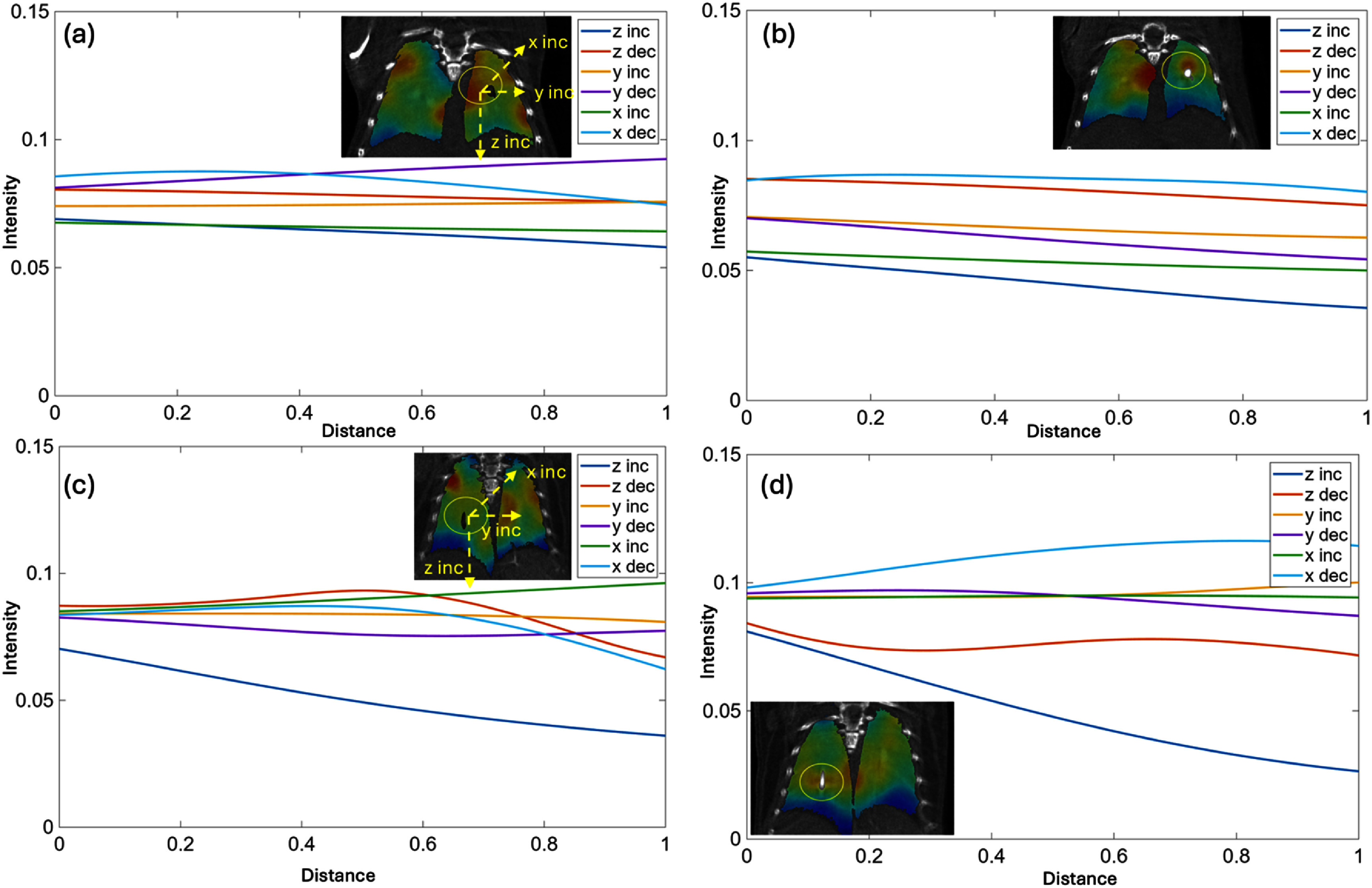
Quantification of the embolization-induced CT-P changes for Case 1 (a) and (b) and Case 2 (c) and (d), measured along nine spatial directions in the axial, coronal, and sagittal planes. For each direction, the mean CT-P intensity (*x*-axis) was computed within rectangular windows positioned at increasing normalized distances (*y*-axis) from the embolus boundary. The horizontal axis represents normalized distance from the embolus, where 0 denotes the embolus boundary, and 1 denotes the furthest point in a given direction. The vertical axis represents mean normalized CT-P intensity. Embolus locations are indicated by yellow circles. Solid and dashed lines distinguish pre- and post-embolization measurements.

Quantifying the spatial relationship between parenchymal perfusion and the embolism represents the first steps toward identifying PE diagnostic criteria from CT-P. In figure 5, we observed two primary indicators that will drive our future investigations. First, the variation in perfusion very near the embolism is a clear marker for the embolism’s position. Second, the decay of perfusion distally from the embolism may aid in identifying affected vasculature and lobes. Since identifying vasculature without a CTPA in humans would require a very high radiation dose to be delivered for high-resolution CT, clot position could be inferred from the CT-P variations. In Case 2, we observe an initial decline in perfusion as we approach the superior portion of the lung, followed by a recovery. This likely reflects crossing from the middle to the superior lung lobe, which has a more normal CT-P. Contrarily, in Case 1, as we approach the inferior portion of the left lung, perfusion does not ever recover. Since the left lung of a mouse is only one lobe, there is no alternative arterial source to recover perfusion distally from the embolism within the same lung.

Finally, we quantified the V/Q difference between our pre- and post-embolism samples. The percentage change in lobe-wise mean CT-V and CT-P can be found in the supplements. V/Q difference was defined as the difference in lobe-wise mean CT-V and CT-P. As can be seen in table 1, for both samples, the largest difference between the change in CT-V and CT-P was in the affected lung lobe. This represents a V/Q mismatch where normal ventilation is co-localized with decreased perfusion, the key diagnostic marker in SPECT PE diagnosis.

**Table 1.** Lobe-wise CT-V/CT-P (V/Q) mismatch percentage changes (%) between pre- and post-embolization for Case 1 and Case 2. Positive values indicate an increase in the V/Q ratio following the embolization, while negative values indicate a decrease. In both cases, the largest absolute change (shown in bold) occurred in the embolized lobe, which is marked by an asterisk (*).

		Right Lung			
	Left lung	*Superior lobe*	*Middle lobe*	*Inferior lobe*	*Post-caval lobe*
Case 1	12.79*	−15.78	8.61	−9.70	2.57
Case 2	9.95	7.38	8.41	13.60*	−0.08

## 4. Discussion

### 4.1. PE alters the blood mass observed in CT-P imaging

This is the first animal study to demonstrate 4DCT-derived perfusion changes before and after pulmonary embolization. Our results indicate that CT-P has the ability to clearly visualize and quantify embolization-induced changes in pulmonary perfusion. Such measurements are not feasible in human subjects. Thus, the animal model allows us to, for the first time, verify the 4DCT-based perfusion imaging method’s ability to capture PE-induced changes in blood mass dynamics. This pilot study also identified spatial heterogeneity in perfusion when comparing healthy lungs to PE lungs, which warrants further study.

We observed decreased perfusion in post-embolism mice, as expected, with perfusion defects at the lung bases downstream of the emboli (figures 3 and 4). Interestingly, although only one lung was embolized in each case, we observed increased perfusion in the contralateral, unaffected lungs. We hypothesize that this may reflect a compensatory redistribution of perfusion in response to the impaired flow in the embolized lungs. However, the interpretation is preliminary, with no inferential statistics given the *n* = 2 sample size, and should further be tested in future studies with larger cohorts. We also hypothesize that this contralateral perfusion increase may be a useful marker for CT-P-derived PE detection, though this requires further validation before any diagnostic conclusions can be drawn. Finally, we observed that perfusion heterogeneity appeared to follow lobe anatomy and lung vasculature, though we note this is a preliminary observation that remains to be confirmed.

For Case 1 (figures 3(b) and 4(b)), the embolus blocks the main intrapulmonary artery in the left lung, where there is only one lobe, and we can see the blood accumulation upstream of the embolus and the perfusion defect downstream. For Case 2, the embolus is blocking the right lung, where there are four lobes (as shown in figures 3(d) and 4(c)), and the embolus seems to be affecting the superior and post-caval lobes. This explains why perfusion distribution differs substantially from Case 1, with a high perfusion region visible as a horizontal red belt in figure 4(c). In addition to higher perfusion upstream of the embolism compared to downstream of the embolism within the middle lobe, we also observed that the unaffected lung lobes had a higher perfusion before embolization than after. The underlying mechanism is still unclear without the ability to precisely trace the vascular tree, and we therefore refrain from further interpretation. Based on our observations, we tentatively hypothesize that differential lobe perfusion could be explored as an additional marker for CT-P-based PE localization in future studies.

Previous SPECT-based approaches diagnose PE by identifying V/Q mismatch, which is defined as reduced perfusion with preserved ventilation. However, this approach does not capture PE-specific hemodynamic changes that can be detected by CT-P imaging. Further, CT-P is not directly comparable to SPECT-P, meaning that an arbitrary cutoff for CT-P intensity would be required to identify a perfusion defect. However, we demonstrated that our CT-V and CT-P methods had sufficient sensitivity to quantify a local mismatch between ventilation and perfusion on a per-sample basis. Further investigation is warranted to identify metrics to diagnose and localize PE without the information provided from the pre-embolization healthy CT images.

### 4.2. Limitations

#### 4.2.1. Sample size

The primary limitation of our study is the limited sample size. We did not perform any statistical analysis or comparisons due to the small sample size (*n* = 2). However, the exploratory nature of this work was aimed to generate hypotheses and guide future studies. All experiments related to induced PE are terminal, and we sought to validate our methodology prior to expanded pre-clinical studies. Future work could include an expanded study to quantitatively validate our findings.

#### 4.2.2. Radiopaque emboli

As our methods depend on differences in HUs to approximate mass change between inhale and exhale CT images, the radiopaque embolizing agent forced us to reduce the computational domain in our CT-P calculation. Our current computational pipeline would not include such high-intensity regions in quantifying CT-P, but we found that the bright emboli prevented our model from avoiding other non-parenchymal structures in the lung. While utilizing radiopaque emboli was necessary to ensure that we could precisely locate the embolism in each case and validate that no breakoff emboli were lodged elsewhere in the lungs, future work with embolizing agents that do not affect image intensities is needed. Such a study would necessitate the inclusion of additional imaging modalities to identify the embolism location, like CTPA, that were not available during the duration of the current study.

#### 4.2.3. Anatomical differences compared to humans

Another limitation of our study is the anatomical differences between mouse and human lungs. Beyond the obvious difference in size between mice and humans, mouse lungs have a different anatomical structure than humans regarding the distribution of lung lobes. While humans have 2 lobes in the left lung and 3 in the right lung, the mouse left lung is one lobe and their right lung consists of 4 lobes. Future studies warrant expansion to animals with a more similar lung structure to humans, like laboratory rats. However, the left lung consisting of one lobe provides useful insight into the single lobe hemodynamics that would not be available in other animals. In the Case 1 mouse, where the embolism is in the left lung, we see the hemodynamic effects of PE to a region of lung tissue that is supplied by a common vascular source. In future studies, we will investigate if the results from Case 1 generalize the hemodynamic effects of acute pulmonary embolization within a single lobe. While there are limited robust approaches to individual lung lobe segmentation, it is an active area of study that may be enhanced by considering the hemodynamics observed in CT-P imaging.

#### 4.2.4. Motion artifacts in 4DCT images

Motion artifacts are a well-recognized challenge in 4DCT imaging and can arise from irregular breathing patterns, anatomical motion occurring in the same or opposite direction as the scanner, and 4DCT binning methods (Carrizales *et al* 2025). In the context of micro-4DCT in mice, these artifacts present a particular limitation, as rodents have substantially higher respiratory rates than humans, making consistent gating challenging (Yuki and Koutsogiannaki 2021), especially for the inhale images. The micro-4DCT images in this study were acquired under free-breathing conditions, and the artifacts were predominantly observed at the bottom of the lungs, manifesting as ‘cold’ regions with lower perfusion following our computational pipeline (figure 3). This is likely due to the movement of diaphragms during free breathing, which creates a transition zone where diaphragm and lung tissue are intermixed in the gated image, making the perfusion estimates in that region unreliable.

Finally, as a pilot study, several methodological uncertainties were not formally assessed, including segmentation reproducibility, the robustness of registration to different parameters, and the sensitivity of CT-P estimates to embolus mask boundaries.

## 5. Future work

### 5.1. Larger cohorts and models that better represent human anatomy

The present pilot study included only two animals, a small sample size that was intentional in order to demonstrate feasibility before expanding to larger cohorts, yet it prevents inferential statistical analysis. Future work should prioritize expanding to larger cohorts to enable hypothesis testing and characterize the variability of CT-P-derived perfusion metrics across subjects. Future work will also focus on expanding our study to animal models whose cardiopulmonary anatomy more closely resembles that of humans. While the mouse model used here was appropriate for initial pilot experimentation, its lobar structure and vascular tree substantially differ from those of humans, limiting the translational relevance. Building on the protocols established in this pilot study, rat models represent a natural next step, offering anatomy more comparable to humans while remaining experimentally tractable.

### 5.2. Non-radiopaque emboli

We primarily sought to study the changes in pulmonary perfusion local to a PE in CT-P imaging. To do so, we used an embolizing agent that would be easily identifiable in CT imaging. We wish to prevent the necessity for alterations to CT-P computation that arise from abnormally bright regions of the image and plan to conduct a similar study with embolisms that do not affect the image intensity in CT. However, as we would still need a method to localize the embolism, we will include additional imaging methodologies like CTPA or magnetic resonance imaging with an MRI detectable embolism (Huynh *et al* 1996). The nonradiopaque beads can be easily fabricated using the same method as the current beads, but without incorporating nanoparticles.

### 5.3. PE classification and localization

A primary focus of future studies should be the development of a computational framework to diagnose and localize PE using CT-P. This study has demonstrated that CT-P is sensitive to the changes induced by PE, and figure 5 presents a spatial CT-P heterogeneity as a potential mechanism to differentiate PE and non-PE lungs. Additionally, machine learning approaches have demonstrated the ability to classify lungs as PE vs healthy but lack explainability and embolism localization. Combining classification models with CT-P for visualization and embolism localization could potentially lead to a computational screening tool for patients at risk for PE.

## 6. Conclusions

While the subset of PE patients with iodine allergy is relatively small, the high fatality rate of PE and the time-critical nature of emergency diagnosis underscore the need for alternative imaging approaches. 4DCT imaging offers a rapid and non-contrast method to capture respiratory dynamics. In this exploratory pilot study with a small pilot cohort, we qualitatively investigated 4DCT-based perfusion imaging in an animal model as a first step toward characterizing its methodological feasibility. Our observations suggest that 4DCT-derived CT-P may be sensitive to embolism-associated hemodynamic changes. These findings support further investigation of 4DCT-derived CT-P as a potential non-contrast approach for PE assessment, pending validation in larger preclinical and clinical studies.

## Data Availability

All data that support the findings of this study are included within the article (and any supplementary information files). Original micro-CT imaging can be made available by request to the corresponding author. Supplementary data available at https://doi.org/10.1088/1361-6560/ae8126/data1.
